# Primary splenic diffuse large B‐cell lymphoma presenting as a splenic abscess

**DOI:** 10.1002/jha2.642

**Published:** 2023-01-24

**Authors:** Paul A. Wadsworth, Roberto N. Miranda, Pooja Bhakta, Peeyush Bhargava, Dylan Weaver, Jianli Dong, Vasily Ovechko, Michael Norman, Palawinnage V. Muthukumarana, Mathew G. Bayes, Jayati Mallick, Kirill A. Lyapichev

**Affiliations:** ^1^ Department of Pathology The University of Texas Medical Branch Galveston Texas USA; ^2^ Department of Pathology Stanford University Stanford California USA; ^3^ Department of Hematopathology the University of Texas MD Anderson Cancer Center Houston Texas USA; ^4^ Department of Internal Medicine The University of Texas Medical Branch Galveston Texas USA; ^5^ Department of Radiology the University of Texas Medical Branch Galveston Texas USA; ^6^ Department of Surgery the University of Texas Medical Branch Galveston Texas USA

**Keywords:** CD30, Splenic B‐cell lymphoma

## Abstract

Diffuse large B‐cell lymphoma (DLBCL) arising in the spleen, also known as primary splenic DLBCL (PS‐DLBCL), is a rare form of malignant lymphoma. It is defined as a lymphoma confined to the spleen or involving splenic hilar lymph nodes. Here we report a case of PS‐DLBCL with CD30. The patient was a 62‐year‐old who presented with 2 weeks of left flank pain, chills, and abdominal distension. Computed tomography identified an 8‐cm splenic mass with central necrosis interpreted as an abscess. A drain was placed, yielding purulent necrotic material; cytologically, only neutrophils were identified. However, purulent drainage continued for 28 days without resolution, prompting splenectomy. Pathological dissection revealed a multinodular mass with central necrosis. Microscopic examination revealed extensive karyorrhexis, abundant ghosts of large cells, and scattered large cells with pleomorphic, multilobated, and vesicular nuclei with moderately abundant cytoplasm. Immunohistochemical staining revealed large, atypical cells positive for CD20, CD30, CD45, PAX5, MYC (>40%), MUM1 (>30%), and p53 (focally). The large cells were negative for CD3 (polyclonal), CD4, CD5, CD8, CD10, CD15, CD34, BCL2, BCL6, AE1/AE3, S100, HHV8, and ALK. The Ki‐67 proliferation rate was approximately 80% in large cells. Notably, this PS‐DLBCL was positive for CD30, an unusual finding among non‐Hodgkin B‐cell lymphomas, which, coupled with the Reed‐Sternberg‐like morphology, raised the possibility of classic Hodgkin lymphoma. Therefore, we reviewed the literature to confirm the unique features of this large B‐cell lymphoma, its abscess‐like appearance, and its expression of CD30.

## 1 INTRODUCTION

Diffuse large B‐cell lymphoma (DLBCL) arising in the spleen, also known as primary splenic DLBCL (PS‐DLBCL), is a rare and poorly characterized form of malignant lymphoma [1]. Although the term has been inconsistently used, primary splenic lymphoma is generally defined as a lymphoma confined to the spleen with common involvement of splenic hilar lymph nodes [2, 3]. PS‐DLBCL may present as a single mass or diffusely intrasinusoidal, suggesting an origin in primary splenic lymphoma. Much more common are secondary splenic lymphomas, where the organ is involved as part of the dissemination of a hematologic neoplasm. The most common lymphoma diagnosis in splenectomy specimens is splenic marginal zone lymphoma (SMZL) [2, 4].

Patients with PS‐DLBCL are typically older (median age, 64 years) and present with left upper quadrant pain, high lactate dehydrogenase levels, and thrombocytopenia [1, 5]. Splenectomy at diagnosis improves survival, especially in patients with early‐stage disease, but it is otherwise treated with chemotherapy similar to systemic forms of DLBCL. Pathologically, PS‐DLBCL is most frequently a large macronodular mass with a sharp transition against an uninvolved spleen. Less frequently, PS‐DLBCL can present with the involvement of white pulp as a micronodular mass, or involving red pulp without a gross mass [5, 6]. PS‐DLBCL can be associated with hepatitis C infection [7]. The top differential diagnoses include systemic DLBCL, T‐cell/histiocyte‐rich large BCL, SMZL, and peripheral T‐cell lymphoma (PTCL), not otherwise specified [5].

Although originally recognized as a marker of classic Hodgkin lymphoma, CD30 is also expressed in a subset of non‐Hodgkin lymphomas, including PTCL, extranodal natural killer/T‐cell lymphomas, cutaneous T‐cell lymphomas, and DLBCL [8]. CD30 positivity in DLBCL, although rare, has been shown to be a favorable prognostic factor [9]. This is in part due to CD30‐positivity enabling treatment with brentuximab‐vedotin, which delivers monomethyl auristatin E, a cytotoxic anti‐microtubule agent to CD30‐positive cells [8].

Here, we present a unique case of PS‐DLBCL which presents as a splenic abscess. Interestingly, this lymphoma expressed CD30. We performed a systematic review of the literature and identified only anecdotal similar cases of PS‐DLBCL and discuss their findings.

### 1.1 Case presentation

**FIGURE 1:**
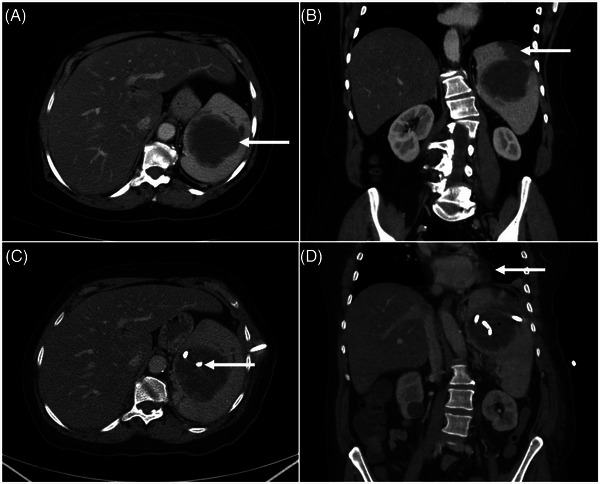
Axial (A) and coronal (B) images from the contrast‐enhanced computed tomography (CT) of the abdomen and pelvis, at the time of the presentation, show a large (∼7 × 7 cm) hypo‐enhancing mass in the spleen (arrow in A) and peripheral wedge‐shaped hypodensity (arrow in B) consistent with an infarct. Follow‐up CT scan (C, D), approximately 3 weeks after placement of the percutaneous drain, shows the pigtail of the catheter inside the lesion (arrow in C) along with a small focus of iatrogenic air (D). No change in the splenic mass and new findings in the left lung base seen as atelectasis and a small pleural effusion (arrow in D)

A 62‐year‐old female presented with left flank pain for two weeks followed by the onset of chills, night sweats, and decreased appetite for 1 week. She was prescribed ciprofloxacin by her primary care provider for suspected upper urinary tract infection; however, negative urine cultures and lack of improvement after 2 days led her to present to the emergency department. On physical exam, she was acutely ill‐appearing with moderate abdominal distension and left‐sided tenderness. Laboratory evaluation demonstrated leukocytosis 25 × 10^9^/L, predominantly mature granulocytes at 21.6 × 10^9^/L), normal platelets at 289 × 10^9^/L, and mild normocytic anemia (Hgb: 11.0 g/dl; Hct: 34.1%; MCV: 88.1 fL). Computed tomography identified splenomegaly with a hypoenhancing area, concerning for an abscess, as well as a wedge‐shaped infarct (Figure 1). Given the appearance of sepsis and splenic abscess, she was initially treated with intravenous antibiotics, and the leukocyte count rapidly returned to normal (9.6 × 10^9^/L). However, the hemoglobin decreased to 9.3 g/dl over the following five days. Patient referred to persistent abdominal pain and developed diarrhea and emesis. After initial surgical evaluation, the patient had a splenic drain placed for purulent material with negative cultures. A fine needle aspiration at that time similarly showed neutrophilic inflammation consistent with an abscess but no malignant cells. During this time the patient was tested positive for severe acute respiratory syndrome coronavirus 2. The patient was discharged, but the tube continued to drain purulent material for 29 days with no change on imaging, prompting a splenectomy.

**FIGURE 2:**
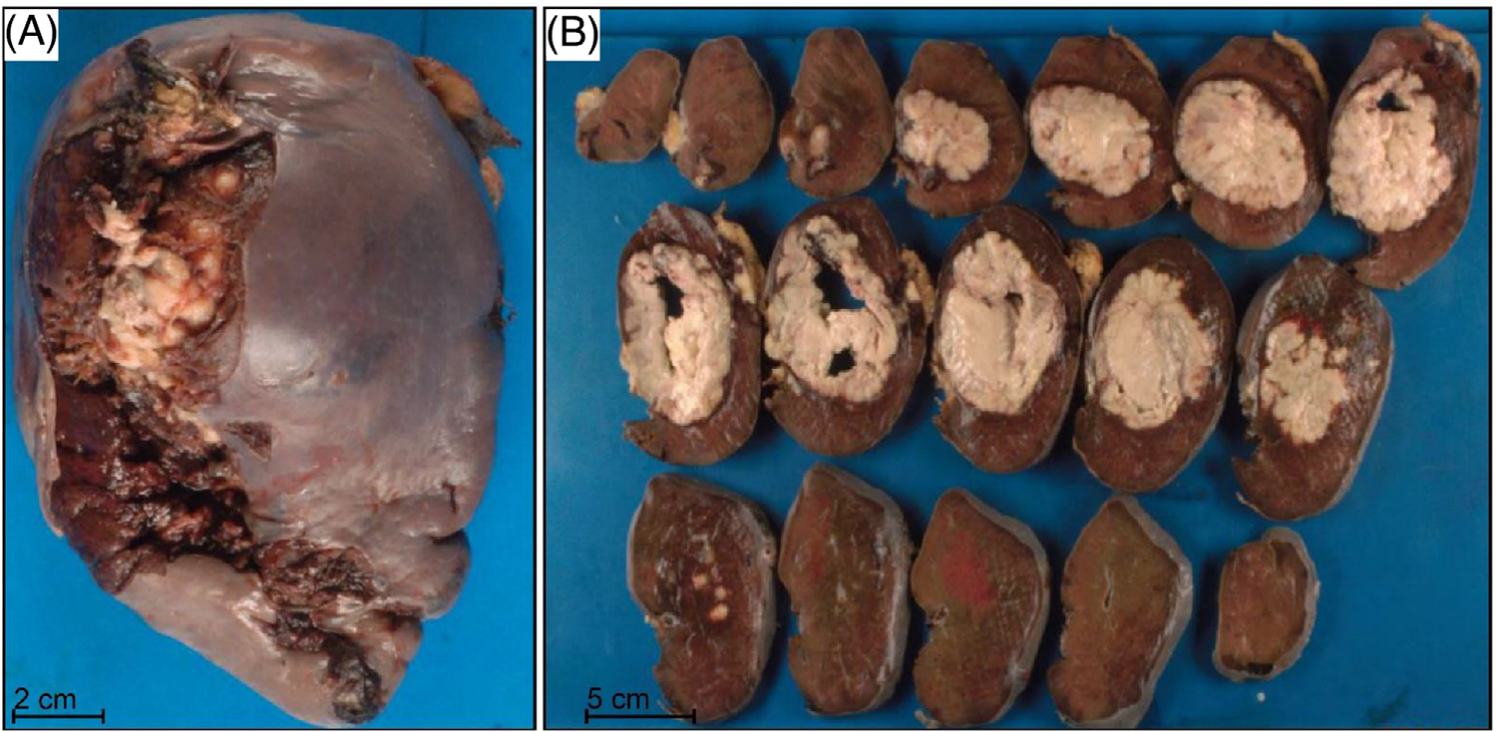
Gross appearance of splenic mass/abscess. (A) The intact splenectomy (14.2 × 9.7 × 7.0 cm, 590 g) revealed a predominantly smooth capsular surface with a focal area of adherent tan‐yellow lobulated adipose tissue and a disrupted area (12.0 × 3.8 cm) located in the hilum. (B) Serial sections reveal a pale tan‐yellow well‐defined soft mass with a central abscess (8.4 × 8.2 × 6.0 cm) which is clearly demarcated from the normal spleen.  There is also a pale‐tan, yellow abscess with surrounding hemorrhage (2.4 × 2.0 × 1.3 cm) underlying the capsular adherent adipose tissue and measuring 0.5 cm from the larger abscess.  The remaining cut surfaces are red‐brown and focally hemorrhagic

Grossly, the dissected spleen appeared to have a centralized pale tan‐yellow well‐defined soft abscess (8.4 × 8.2 × 6.0 cm) disrupting the hilum, as well as an additional pale‐tan yellow abscess with surrounding hemorrhage (2.4 × 2.0 × 1.3 cm) measuring 0.5 cm from the larger abscess (Figure 2). Similar to as previously reported for the macronodular subtype of PS‐DLBCL [5], there was a sharp transition between the mass and uninvolved spleen. The remainder of the spleen was focally hemorrhagic.

**FIGURE 3:**
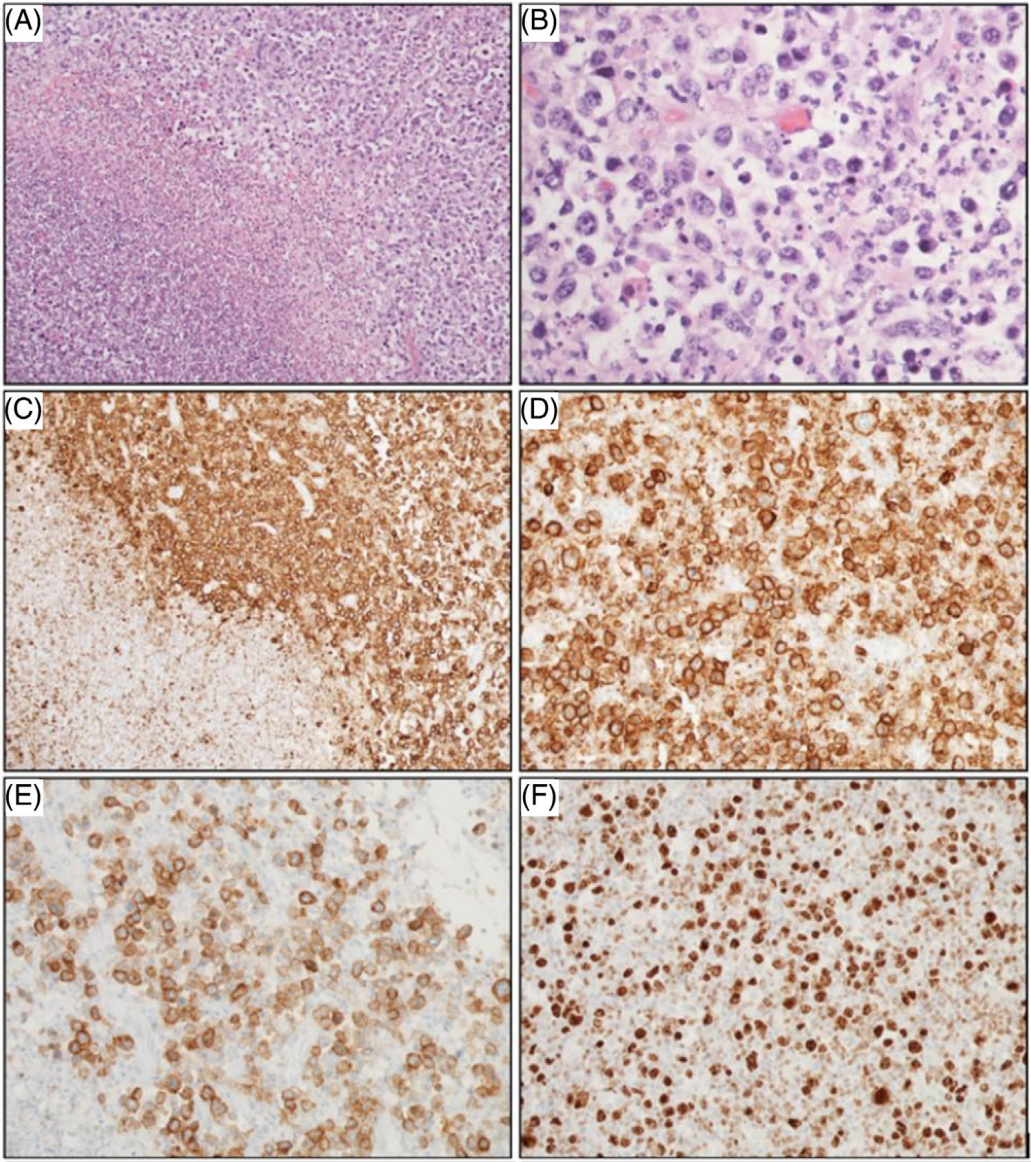
Histology and immunohistochemistry of primary splenic diffuse large B‐cell lymphoma (PS‐DLBCL). (A, B) Focally necrotic splenic tissue is infiltrated by large, atypical lymphoid cells shown by H&E ([A] 100X; [B] 400X). The atypical cells demonstrate predominantly centroblastic morphology with round to oval nuclei, fine chromatin, multiple small nucleoli, and moderate amounts of cytoplasm. By immunohistochemistry, CD20‐positive neoplastic cells surround a central area of necrosis ([C] 100X); at higher power, significant size variability among CD20‐positive atypical cells is observed for CD20 ([D] 200X) and CD30 ([E] 200X). [F] Malignant PS‐DLBCL cells demonstrated a high proliferative index with Ki‐67 expression of ∼80% (200X)

Histologic sections revealed fragments of focally necrotic splenic tissue infiltrated by large, atypical lymphoid cells (Figure 3 A,B). The atypical cells had predominantly centroblastic morphology with round to oval nuclei, fine chromatin, multiple small nucleoli and moderate amounts of cytoplasm. Numerous apoptotic bodies and mitoses were seen.

Immunohistochemical stains on fixed, paraffin‐embedded tissue sections demonstrated that the large atypical lymphoid cells were positive for CD20 (Figure 3 C,D), CD30 (Figure 3E), CD45, PAX5, cMYC (>40%), MUM1 (>30%), and p53 (focally). The large cells were negative for CD3P, CD4, CD5, CD8, CD10, CD15, CD34, BCL2, BCL6, AE1/AE3, S100, HHV8, and ALK. CD3P highlighted small infiltrating T‐cells. The Ki‐67 immunostain demonstrated a proliferative index of approximately 80% (Figure 3F). In situ hybridization (ISH) for Epstein‐Barr virus (EBV) (EBER stain) and for kappa and lambda light chains were negative. Fluorescence in ISH studies was positive for rearrangement (break‐apart) of *BCL6* (3q27), but negative for *MYC* rearrangement (8q24 locus) or double fusion of t(14;18) *IgH* (14q32) with *BCL2* (18q21).

**FIGURE 4:**
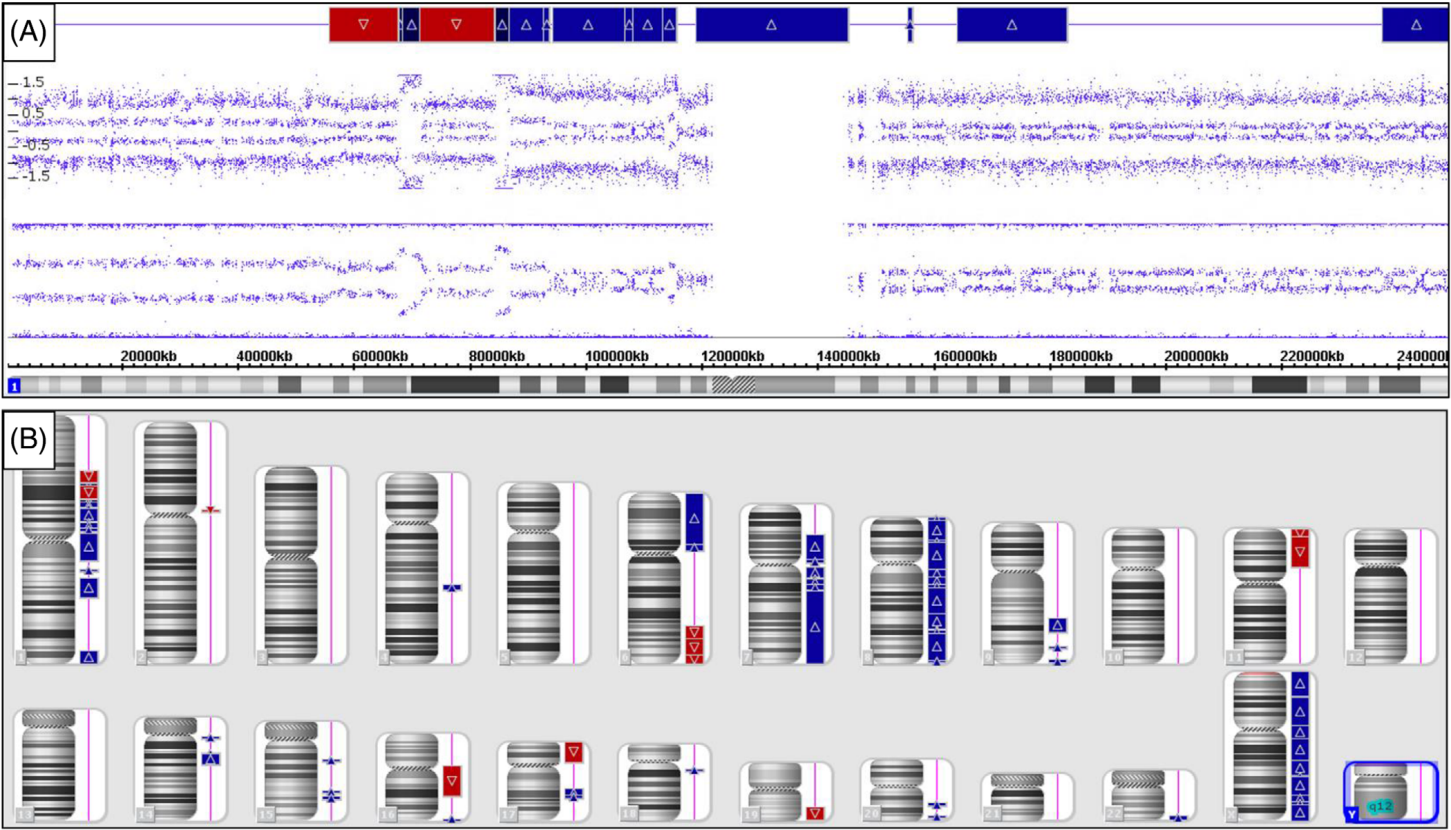
Chromosome microarray analysis of neoplastic cells identified complex DNA copy number aberrations. (A) There are mosaic losses (in 1p) and gains (in 1p and 1q) of chromosome 1, with copy number changes consistent with chromothripsis. (B) Chromosomes depicted by Karyoview with gains shown in blue and losses in red. There are segmental losses in 6q, 11p, 16q, and 17p, and gains in chromosomes 6p, 7, 8, and X. These changes may be useful to monitor clonal evolution and progression of the lymphoma cells

Additionally, a chromosome microarray was performed at the University of Texas Medical Branch and was consistent with complex DNA copy number aberrations. There are mosaic losses (in 1p) and gains (in 1p and 1q) of chromosome 1, with copy number changes consistent with chromothripsis (Figure 4A). There are also segmental losses in 6q, 11p, 16q, and 17p and gains in chromosomes 6p, 7, 8, and X (Figure 4B). Based on morphologic features and immunohistochemical analysis, the diagnosis of PS‐DLBCL was established. The primary care physician was contacted, and the diagnosis was communicated. The patient is currently under the care of an oncologist at an outside institution.

## 2 MATERIALS AND METHODS

### 2.1 Histology and immunohistochemistry

Hematoxylin and eosin and immunohistochemical (IHC) stains were performed on formalin‐fixed, paraffin‐embedded tissue sections at the University of Texas Medical Branch.

The following IHC stains were used: CD4, CD5, CD8, CD10, CD34, PAX‐5, and MUM‐1 (Agilent/Dako, Carpinteria, California); CD3P, CD15, CD20, CD30, AE1/AE3, BCL‐2, Ki‐67 (Ventana/Roche, Tucson, Arizona); CD45, BCL‐6 and c‐MYC (Cell marque, Rocklin, California); P53 and S100 (Ventana/RTU); HHV8 (Cell Marque/RTU); and ALK‐1/CD246 (Dako/RTU). ISH was performed using a probe for EBV‐encoded viral RNAs (EBER, Ventana/Roche, Tucson, Arizona).

### 2.2 Cytogenetics and fluorescence ISH

Fluorescence ISH analysis was performed by Vitro Molecular (Miami, FL) with probes (Abbott Molecular/Vysis and Cytocell) specific for *BCL6* (3q27), *MYC* (8q24), and t(14;18) *IgH/BCL2* was performed on fresh tissue.

### 2.3 Chromosomal microarray

Chromosomal microarray analysis was performed using Affymetrix CytoScan HD microarray. This microarray and associated software (Chromosome Analysis Suite) are manufactured by Affymetrix (Santa Clara, CA) and used by the University of Texas Medical Branch Molecular Diagnostics Laboratory (MDL) for the purpose of identifying DNA copy number gains and losses associated with large chromosomal imbalances.[10]

## 3 DISCUSSION

Given that primary splenic lymphoma constitutes ≤1% of all malignant lymphomas, consistent and detailed characterization is lacking [1, 2]. Few case series containing immunohistochemistry on primary splenic lymphoma have been published (none solely on PS‐DLBCL) [3, 4, 6, 11]. Much more commonly, splenic involvement in lymphoma occurs secondarily. When a primary splenic BCL is suspected, top differentials to explore include systemic DLBCL, T‐cell/histiocyte‐rich large BCL, SMZL, and PTCL.

Genetic testing for PS‐DLBCL typically reveals monoclonal *IGH* rearrangements, along with cytogenetic abnormalities including add(7p22), del(8p22), add(19p13), and t(3;6) [5]. However, we observed only *BCL6* rearrangement, and our cytogenetic testing revealed changes not previously reported, including chromothripsis of chromosome 1, segmental losses in 6q, 11p, 16q, and 17p, and gains in chromosomes 6p, 7, 8, and X. These changes may be useful to monitor clonal evolution and progression of splenic lymphoma.

Of particular interest is the presentation of this splenic lymphoma as a continuously‐draining abscess. In the literature review, we identified four reports of splenic lymphoma presenting as an abscess: marginal zone lymphoma [12], DLBCL [13, 14], and Hodgkin lymphoma.[15] Most similar to our patient was the case of the marginal zone lymphoma; percutaneous drainage, in that case, yielded purulent material that grew *Staphylococcus aureus* and *Klebsiella pneumoniae*, but the patient failed to significantly improve with antibiotics. The negative splenic cultures following drainage may have been due to antibiotic treatment (i.e., an infected abscess within the PS‐DLBCL was sterilized prior to drainage), or the splenic abscess may have been ancillary with a simultaneous infection at another site (i.e., urinary tract) at presentation.

Regardless, the marked leukocytosis and rapid improvement following IV antibiotics suggest infection in our patient at presentation. Additionally, we speculate that purulent splenic abscess could play an etiological role in PS‐DLBCL development, and more cases need to be studied and described to make it a more prominent conclusion.

## 4 CONCLUSION

In conclusion, we report a striking and unusual case of PS‐DLBCL presenting as a splenic abscess that continuously drained sterile purulent material for 28 days. The literature review demonstrated very rare cases of splenic lymphomas presenting as an abscess, therefore we wanted to highlight the importance of keeping DLBCL as a differential in such a presentation.

## AUTHOR CONTRIBUTIONS

Paul A. Wadsworth and Kirill A. Lyapichev wrote the manuscript with support from Roberto N. Miranda, Peeyush Bhargava, Dylan Weaver, Michael Norman, Jianli Dong, Vasily Ovechko, Palawinnage V. Muthukumarana, Mathew G. Bayes, and Jayati Mallick. All authors discussed the results and contributed to the final manuscript.

## CONFLICT OF INTEREST

The authors declare that they have no conflict of interest.

## FUNDING INFORMATION

The authors received no financial support for the research, authorship, and/or publication of this article.
